# Coverage of Jade Goody's cervical cancer in UK newspapers: a missed opportunity for health promotion?

**DOI:** 10.1186/1471-2458-10-368

**Published:** 2010-06-24

**Authors:** Shona Hilton, Kate Hunt

**Affiliations:** 1 MRC Social and Public Health Sciences Unit, 4 Lilybank Gardens, Glasgow, UK

## Abstract

**Background:**

It has been claimed that publicity surrounding popular celebrity Jade Goody's experience of cervical cancer will raise awareness about the disease. This study examines the content of newspaper articles covering her illness to consider whether 'mobilising information' which could encourage women to adopt risk-reducing and health promoting behaviours has been included.

**Methods:**

Content analysis of 15 national newspapers published between August 2008 and April 2009

**Findings:**

In the extensive coverage of Goody's illness (527 articles in the 7 months of study) few newspaper articles included information that might make women more aware of the signs and symptoms or risk factors for the disease, or discussed the role of the human papilloma virus (HPV) and the recently introduced HPV vaccination programme to reduce the future incidence of cervical cancer. For example, less than 5% of articles mentioned well-known risk-factors for cervical cancer and less than 8% gave any information about HPV. The 'human interest' aspects of Goody's illness (her treatment, the spread of her disease in later months, her wedding, and her preparations for her children's future) were more extensively covered.

**Conclusions:**

Newspaper coverage of Goody's illness has tended not to include factual or educational information that could mobilise or inform women, or help them to recognise early symptoms. However, the focus on personal tragedy may encourage women to be receptive to HPV vaccination or screening if her story acts as a reminder that cervical cancer can be a devastating and fatal disease in the longer term.

## Background

On the 22^nd ^of March 2009, reality television star Jade Goody died from cervical cancer aged 27 years. Her seven-month battle with cancer attracted intense media coverage across the world. In the UK, this publicity coincided with the introduction in September 2008 of the Human Papillomavirus (HPV) vaccination programme aimed at protecting girls from cervical cancer. The timing of the coverage of Goody's experience of cervical cancer is thus potentially crucial as uptake of vaccines depends on perceptions of the balance between any putative adverse effects of a vaccine and the perceived severity and consequences of contracting the disease, [1-4] both of which can be shaped by popular media coverage[5,6]. It has been widely claimed that Goody's illness has led to greater awareness of the disease and increased uptake of cervical cancer screening, the so-called 'Goody Effect'. On the day of her death Britain's Prime Minister, Gordon Brown, said in a press statement that: "her family can be extremely proud of the work she has done to raise awareness of cervical cancer which will benefit thousands of women across the UK"[7].

On a global scale, cervical cancer represents the second most frequent cancer in women, and kills 950 women each year in the UK[8]. Established risk factors for cervical cancer include: early sexual intercourse, number of sexual partners (and the number of sexual partners that each partner has had), smoking and a family history of cervical and ovarian cancer[9,10]. Since the 1970s it has been known that HPV is the major cause of cervical cancer[11]. HPV infection is very common; it is estimated that 20% of sexually active girls will contract the virus by the age of 18 years, [12] and up to 80% of all sexually active women are likely to be affected with at least one virus type in their lifetime[13]. However, only a small proportion of women who encounter persistent infection from high risk genotypes (HPV 16 and 18, and a few other strains [14,15]) go on to develop cervical cancer; 90% of infections resolve spontaneously[16,17]. Public awareness of HPV as a risk factor lags behind these scientific discoveries. In a study of a representative sample of British women (n = 1620) in 2006-7, only 2.5% cited HPV as a cause of cervical cancer. Awareness of HPV was lowest in those with least formal education and from lower income households,[18] that is precisely the groups who are at highest of the disease (e.g. research in Scotland found that cervical cancer rates amongst women living in the most deprived areas were more than two and a half times those of women in the least deprived areas [19]).

Women from disadvantaged backgrounds are also least likely to utilise cervical screening services [20] or to be aware of the risk factors associated with cervical cancer. It has been suggested that popular media could be employed to raise public awareness about HPV and its links with cervical cancer, [21] and to encourage women to be receptive to HPV vaccination and cervical cancer screening. However, it has long been recognised that information in the media can increase social inequalities in knowledge [22] and there is criticism that, when reporting health risks, the news media often omit 'mobilising' information that, in theory, allows readers to act on existing attitudes[23]. Thus, it would seem that the 'Goody Effect' could only contribute to an appreciable 'saving' of lives if the mass media provide mobilising information that encourages those most at risk to adopt risk-reducing and health promoting behaviours.

This study examines the information about cervical cancer included in newsprint media coverage of Jade Goody's illness and death. Her story is of interest both because she was clearly identified in the media as coming from a less advantaged background (and thus perhaps being a role model for women at greatest risk from cervical cancer) and because of her prominence in the popular press over the preceding few years. She had been the target of ridicule and vilification in the UK press for her lack of general knowledge and after appearing drunk and naked on the 'reality' television series *Big Brother 3 *[24]. After leaving the Big Brother house she continued to attract media attention including allegations of racist bullying whilst participating in *Celebrity Big Brother *. In August 2008, whilst participating in the Indian version of *Celebrity Big Brother*, she received her diagnosis of cervical cancer on camera and returned to the UK for treatment where she remained in the media spotlight until her death. Her story thus has great potential to inform some sections of the British public about the risks, prevention and management of cervical cancer, and to reinforce or challenge existing images of cancer more generally. The framing of media stories around such 'human interest' stories can be a powerful means of engaging audiences, and can influence what it included or excluded from coverage[25,26].

Given the widespread claims that coverage of Goody's illness will raise awareness of cervical cancer and 'save lives', our aim in this paper is to report a content-analysis of newspaper articles about Goody's illness. This sought to document the extent to which information about cervical cancer (e.g. information on risk factors, background statistics, signs and symptoms of the disease) and cervical screening were included in coverage.

## Methods

### Newspaper selection

We selected 15 UK newspapers with high circulation figures and a range of readership profiles http://www.abc.org.uk, http://www.nrs.co.uk, including 12 UK national newspapers and 3 Scottish newspapers. The sample comprised 8 'serious' newspapers (*Times*, *Guardian*, *Telegraph*, *Independent*, *Sunday Times*, *Observer, Herald, Scotsman *), two 'middle-market tabloid' (*Daily Mail, Express *) and five 'tabloid' newspapers (*Daily Record*, *Mirror*, *Sun*, *Sunday People*, *News of the World *) http://www.nmauk.co.uk/nma/do/live/marketPlaceCharts. This typology has been used by others to select newspapers with a range readership profiles and political orientations[27]. It is argued that UK newspapers are well suited to studies which aim to differentiate mass media discourses by intended readers' age and social class because the UK newspaper market is powerfully segmented into 'tabloid' and 'serious' genres catering for distinct readership groups. For example, *The Sun *and *Daily Mirror *tend to be read by a younger readership from lower social class backgrounds, the *Telegraph *and *The Times *tend to have an older readership from more affluent backgrounds, and the *Daily Mail's *intended readership is women[28].

### Search strategy

Our search period was from 19^th ^August 2008, the date on which Jade Goody received her diagnosis of cervical cancer, to Sunday 5^th ^April 2009, the day after her funeral. Articles in all 15 newspapers were identified using the *Newsbank *archival database, an online resource which enables the content of various newspapers to be electronically searched (see http://www.eastsussex.gov.uk/libraries/reference/newsbank.htm. The search terms used in 'All Text' were: Jade Goody and cervical; Jade Goody and HPV; Jade Goody and humanpapilloma; Jade Goody and Human papilloma; and, Jade Goody and vaccin*. All articles types were included with the exception of letters. The search identified 631 articles. These articles were screened to identify and remove any duplicates or articles published in Irish editions. After this screening 537 articles remained for analysis.

### Data Analysis

A coding frame was developed through an iterative process. Thirty randomly selected articles were examined (SH) to identify the key discourses; these became thematic categories in the initial coding frame. This was tested against a further randomly selected 25 articles (every twentieth article in the sample) and revisions were made to the coding frame. Once there was no further identification of thematic categories two researchers (SH, KH) coded the entire sample of articles together, resolving any queries by discussion on an ongoing basis. The final coding framework recorded the newspaper in which the article appeared, publication date, headline, word count, and whether there was an image. The broad thematic categories focus on six key topic areas in order to assess whether the mass media provide mobilising information on: HPV infection; HPV vaccination; information about cervical cancer; risk factors associated with cervical cancer; information about cervical screening; and Jade Goody's experience of cervical cancer.

In order to systematically quantify the content of the articles, each article was read line by line and coded to indicate whether or not each of the thematic categories in the coding frame was mentioned. We coded for manifest content[29]. Manifest content refers to what is explicitly stated and thus draws on the objective and replicable qualities of quantitative methods[30]. The data were entered into SPSS 14. We assessed whether the broad thematic categories were mentioned to a similar extent in the three genres of newspapers by crosstabulating the themes, one by one, by genre of newspaper. Chi-square statistics were calculated in SPSS (the significance level was set at p < 0.05).

## Findings

Between 19^th ^August 2008 and 5^th ^April 2009 527 news articles which satisfied the inclusion criteria were published in these 15 newspapers, demonstrating the apparent newsworthiness of 'celebrity cancer'. Three quarters (73.1%, 385/527) were published in 'serious' newspapers, one fifth in 'tabloid' newspapers (19.9%, n = 105) and 7.0% (n = 37) in 'middle-market tabloids' (see table 1). The average word count was 787 words for 'middle-market tabloid' articles, 675 words for 'tabloid' articles and 578 words for 'serious' newspaper articles.

**Table 1:** Articles (n = 527) by newspaper genre and publication

Genre	Title of publication	No. of articles (%)
**Serious**	Times	20 (3.8)
	Guardian	29 (11.2)
	Telegraph	8 (1.5)
	Independent	11 (2.1)
	Scotsman	41 (7.8)
	Herald	46 (8.7)
	Sunday Times	108 (20.5)
	Observer	122 (23.1)
	
	**385 (73.1)**	

**Middle-market tabloid**	Daily Mail	22 (4.2)
	Express	15 (2.8)
	
	**37 (7.0)**	

**Tabloid**	Mirror	11 (2.1)
	Sun	20 (3.8)
	Daily Record	19 (3.6)
	News of the World	20 (3.8)
	Sunday People	35 (6.6)
	
	**105 (19.9)**	

**Figure 1:**
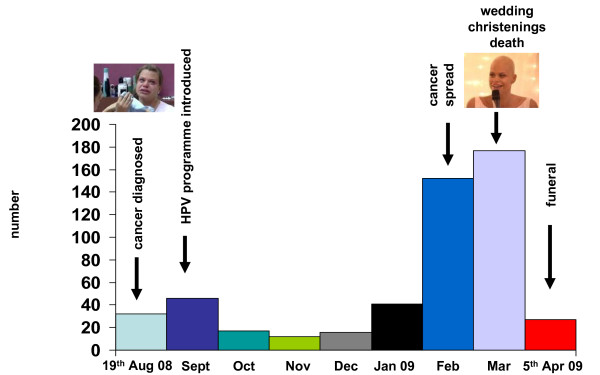
Aligning key events in Jade Goody's cancer story with newspaper articles (n=527)

Figure 1 aligns key events in Goody's cancer story with the total number of articles month by month over the study period and shows a marked increase in the number of articles in February 2009 after her cancer was reported to have spread and become terminal. The largest number of articles appeared in March 2009 when she married, was christened alongside her two young children, and died, again emphasizing the power of the 'human interest' side of such stories.

### Key issues raised in the coverage

Some topics received far more attention than others. Across the newspaper genres, articles were much more likely to cover personal details of Goody's story than to include more factual, educational information on HPV, cervical cancer and cervical screening (see table 2). Over 40% of the articles included mention that her illness was terminal (40.8%), around a third of articles reported on her treatment (35.5%, including her chemotherapy and alopecia following this, and her surgery) or her diagnosis (31.5%). Her experience of pain or pain management was mentioned in one fifth of articles (19.4%). Much of the later coverage was about decisions she made following her diagnosis: around one fifth reported her wedding (20.9%), a tenth reported on the christenings (12.0%) and her funeral (11.8%), and many articles reported on her preparations for her children's future financial security (27.3%) or psychological well-being (13.9%). For example, it was reported that she gave her sons wristbands to rub when they missed her (5th April 09, *News of the World *), left them a memory box with a collection of personal belongings (29th Mar 09, *Daily Record *), and told them:

**Table 2:** Key themes in articles, overall (n = 527) & by newspaper genre

		Newspaper genre (n, column %)			
**Themes**	_**N articles (% of 527)**_	_**Serious (% of 385)**_	_**Middle-tabs (% of 37)**_	_**Tabloids (% of 105**_	_**X**_^**2 **^**(2 df) (p value)**

**HPV information**					

Causes cervical cancer	_40 (7.6)_	_26 (6.8)_	_7 (18.9)_	_7 (6.7)_	_7.28 (0.03)_
Is a STI	_24 (4.6)_	_13 (3.4)_	_5 (13.5)_	_6 (5.7)_	_8.39 (0.02)_
Can cause genital warts	_5 (0.9)_	_2 (0.5)_	_1 (2.7)_	_2 (1.9)_	_2.30 (0.23)_
Is common (statistics HPV rates)	_9 (1.7)_	_5 (1.3)_	_2 (5.4)_	_2 (1.9)_	_3.42 (0.18)_
Can resolve on its own and not progress to cancer	_8 (1.5)_	_5 (1.3)_	_2 (5.5)_	_1 (1.0)_	_4.09 (0.13)_

**HPV vaccination**					

Details of programme guidelines	_22 (4.2)_	_13 (3.4)_	_6 (16.2)_	_3 (2.9)_	_14.48 (0.001)_
Any advocacy of HPV vaccination	_42 (8.0)_	_24 (6.2)_	_9 (24.3)_	_9 (8.6)_	_15.13 (0.001)_
Any concerns about adverse reactions to HPV vaccination	_3 (0.6)_	_2 (0.5)_	_0_	_1 (1.0)_	_-_

**Cervical cancer**					

Background statistics	_50 (9.5)_	_36 (9.4)_	_8 (21.6)_	_6 (5.7)_	_8.09 (0.02)_
Signs and symptoms	_15 (2.8)_	_11 (2.9)_	_0_	_4 (3.8)_	_1.44 (0.49)_

**Risk Factors: Cervical cancer**					

Socio-economic background	_8 (1.5)_	_7 (1.8)_	_0_	_1 (1.0)_	_1.03 (0.60)_
Default on cervical screening	_20 (3.8)_	_18 (4.7)_	_0_	_2 (1.9)_	_3.30 (0.19)_
Number of sexual partners	_15 (2.8)_	_8 (2.1)_	_2 (5.4)_	_5 (4.8)_	_3.09 (0.21)_
Age at first sexual intercourse	_12 (2.3)_	_8 (2.1)_	_2 (5.4)_	_2 (1.9)_	_1.76 (0.42)_
Smoking	_8 (1.5)_	_5 (1.3)_	_1 (2.7)_	_2 (1.9)_	_0.58 (0.75)_

**Cervical Screening**					

Cervical cytology advocacy	_2 (0.4)_	_2 (0.5)_	_0_	_0_	_-_
Cervical smear uptake rates	_78 (14.8)_	_54 (14.0)_	_11 (29.7)_	_13 (12.4)_	_7.21 (0.03)_
Cervical screening guidelines	_34 (6.5)_	_30 (7.8)_	_1 (2.7)_	_3 (2.9)_	_4.26 (0.12)_
Debate about lowering the age of screening in England	_59 (11.2)_	_44 (11.4)_	_3 (8.1)_	_12 (11.4)_	_0.38 (0.83)_

**Aspects of Jade Goody's cancer 'story'**					

Her diagnosis with cervical cancer	_166 (31.5)_	_132 (34.3)_	_11 (29.7)_	_23 (21.9)_	_5.92 (0.05)_
Her previous abnormal smear results	_38 (7.2)_	_29 (7.5)_	_1 (2.7)_	_8 (7.6)_	_1.21 (0.55)_
Her cancer treatment (chemo, operations etc)	_187 (35.5)_	_138 (35.8)_	_6 (16.2)_	_43 (41.0)_	_7.39 (0.03)_
The spread of her cancer/cancer diagnosed as terminal	_215 (40.8)_	_153 (39.7)_	_28 (75.7)_	_34 (32.4)_	_21.89 (<0.001)_
Pain management	_102 (19.4)_	_71 (18.4)_	_8 (21.6)_	_23 (21.9)_	_0.77 (0.68)_
Preparing for her children's financial future	_144 (27.3)_	_97 (25.2)_	_21 (56.8)_	_26 (24.8)_	_17.37 (<0.001)_
Preparing children's psychologically wellbeing	_73 (13.9)_	_52 (13.5)_	_2 (5.4)_	_19 (18.1)_	_3.84 (0.147)_
Coverage of her wedding	_110 (20.9)_	_69 (17.9)_	_19 (51.4)_	_22 (21.0)_	_22.84 (<0.001)_
Coverage of christenings	_63 (12)_	_38 (9.9)_	_11 (29.7)_	_14 (13.3)_	_12.89 (0.002)_
Planning time after death	_63 (12)_	_39 (10.1)_	_6 (16.2)_	_18 (17.1)_	_4.54 (0.10)_
Coverage of her funeral	_62 (11.8)_	_45 (11.7)_	_6 (16.2)_	_11 (10.5)_	_0.88 (0.65)_
Mentions of her story as raising awareness of cervical cancer	_196 (37.2)_	_135 (35.1)_	_21 (56.8)_	_40 (38.1)_	_6.85 (0.03)_

"Mummy's going to heaven soon...I'm going to be a star up in the sky so when you are looking up you will be able to see me and know I'm there, always looking over you. ... Close your eyes and you can talk to me." (15th Mar 09, *The Sun *).

More than a third of articles (37.2%) made some claim that her illness and untimely death would save lives by raising public awareness of cervical cancer. Typical headlines stated that the "Goody effect is a legacy that will save other people's lives" (23^rd ^Mar 09, *Mirror *) and that "Jade effect is making us all aware" (14^th ^Mar 90, *Herald *).

However, 'mobilising' information about risk behaviours or risk factors for the development of cervical cancer was rarely included. For example, the detrimental impact of defaulting on screening was mentioned in 20 articles (3.8%), having multiple sexual partners in just 15 articles (2.8% and only mentioned in relation to women), early age at first sexual intercourse in 12 articles (2.3%), and deprivation and smoking in 8 articles each (1.5%). Statistics on cervical cancer prevalence rates were reported in less than a tenth of articles (9.5%) and the signs and symptoms of cervical cancer were mentioned in just 15 articles (2.8%).

Information that might encourage women to adopt health promoting behaviours in relation to cervical screening was also assessed. Uptake rates were mentioned in 14.8% of articles and the debate about the discrepancy in cervical screening policy in England and Scotland appeared in 11.2% of articles. Cervical screening guidelines were given in 34 articles (6.5%) and cervical smears were explicitly advocated in only two articles, although their importance was inferred in 13.9% of articles.

Despite the introduction of the HPV vaccination programme in the UK in September 2008 very shortly after Goody's diagnosis, information about HPV and its role in cervical cancer was seldom included. Only 40 articles mentioned that HPV causes cervical cancer (7.6%) and only 24 that HPV is a sexually transmitted infection (4.6%). The fact that HPV is a very common infection was reported in just 9 articles (1.7%), can resolve on its own in 8 articles (1.5%) and can cause genital warts in 5 articles (0.9%). HPV vaccination was advocated in just 42 articles (8.0%) and guidelines about the HPV vaccination programme were given in only 22 articles (4.2%). However, concerns about the safety of vaccination were also largely absent (3 articles related a story about a girl who allegedly became paralysed in one leg following HPV vaccination).

### Comparison of the coverage in different newspaper genres

There was surprisingly little difference between the themes covered in the newspapers of different genres (see right-hand columns of table 2). For most of the themes considered no differences were apparent; where there was an indication that coverage of themes differed (taken at conventional levels of significance, p < 0.05), it was articles in the 'middle market tabloids' that tended to differ from those in the 'serious' and 'tabloid' newspapers. With respect to cervical cancer information, the 'middle market' tabloids were much more likely to mention details of the HPV vaccination programme (p = 0.001), advocacy for vaccination (p = 0.001), and to a lesser extent the causes of cervical cancer (p = 0.03), that HPV is sexually transmitted (p = 0.02), background statistics on cervical cancer (p = 0.02) and cervical smear uptake rates (p = 0.03). With respect to Goody's own 'cancer story', the 'middle market' tabloid articles were more likely to report that her cancer had spread (p < 0.001), how she was preparing for her children's future finances (p < 0.001), her wedding (p < 0.001) and christening (p = 0.002), and that her experience would raise awareness of cervical cancer (p = 0.03). 'Serious' and 'tabloid' newspapers were more likely to include coverage of her treatment (p = 0.03).

## Discussion

The mass media has been described as "the nexus between public and policy agenda" and as "highly influential in shaping discourses about health and research", although it is acknowledged that how news media affect public perceptions of diseases is "complex and diverse" (p569)[6]. Although it has been widely claimed that newspaper publicity surrounding Jade Goody's cervical cancer will raise awareness and 'save lives', this study found that relatively few newspaper articles included the kind of mobilising information that could inform women how best to manage their risk for HPV and cervical cancer. Analyses of the impact of other high profile celebrity illnesses suggest that, in the short-term at least, the 'Goody effect' may act as a powerful incentive to utilise cervical screening services[31-35]. Indeed, despite the trend towards declining uptake rates for cervical screening over the last decade in the UK, cervical screening uptake figures for both England and Scotland for 2008-09 suggest that there has been a slight increase in coverage, particularly among younger age groups. The cervical screening figures for England published on 31^st ^March 2009 suggest that the proportion of 25 to 49 year olds (screened every 3 to 3.5 years) increased to 72.5% compared with 69.3% in the previous year[36] The published data in Scotland also suggest an increase, with the largest increase in uptake amongst younger women aged 20-24 years[37]. Whilst some reports of a 20% increase in uptake need to be interpreted with caution due to changes to the Scottish recording system for collecting data,[38] these data suggest a reversal in the trend towards declining uptake which could in part be attributable to media coverage of Goody's illness.

The diagnosis and experience of illness amongst other celebrities, such as Olivia Newton John (breast cancer), Lance Armstrong (testicular cancer), Michael J Fox (Parkinson Disease) and Katie Couric (Colon Cancer) have also been credited with raising public awareness about their illnesses[31-34]. However, heightened visibility of a celebrity's illness can produce less desirable reactions from a public health and cancer education standpoint. For example, the popstar Kylie Minogue's diagnosis with breast cancer was associated with a 20% increase in breast imaging procedures in Australia in the first and second quarters after publicity about her diagnosis, but the volume of operations to excise breast cancer did not change, suggesting that the publicity caused some women to undergo unnecessary interventions and raised levels of anxiety[35]. Similarly, a UK survey of 2289 women conducted after Minogue's diagnosis found that 77% of women thought that the risk of breast cancer is higher in women below the age of 70. The authors concluded that the 'Kylie Effect' led young women to panic, increasing demands for breast screening, whilst misleading older women (who were most at risk) to think that they were less at risk of breast cancer[39]. Thus, it seems that cancer stories in the popular media often focus on 'human interest' aspects at the expense of educational messages about specific cancers or cancer in general.

In the articles covering Jade Goody's cervical cancer story there was almost no mention of her sexual history or behaviour. The media's coyness about discussing the association between cervical cancer risk and sexual behaviour in these articles is in stark contrast to the negative scrutiny of Goody's personal life prior to her diagnosis with cancer. It also contrasts with recent newspaper coverage of cervical cancer 'framed' [25] around the introduction of the HPV vaccination programme in the UK. In the HPV coverage newspapers commonly highlighted how women's sexual behaviours affected the risk of transmission of HPV, potentially reinforcing notions that women are responsible for sexual contagion and double-standards about sexual activity in young men and women[26]. From a public health and cancer education perspective the difference in the focus of stories covering Goody's cervical cancer compared with newspaper coverage of HPV vaccination is perhaps all the more surprising given that they were two news stories linked to cervical cancer published in the same 15 newspapers, running over some of the same period. These differences illustrate how the 'framing' of stories causes potentially pertinent information to be included or excluded.

The coverage of Goody's illness focussed more on one young woman's experience of diagnosis, treatment and death from cervical cancer and is likely to contribute to a general public awareness that cervical cancer can be a devastating and fatal disease. This knowledge could mobilise women to take action and encourage them to be more receptive to screening or HPV vaccination, as research on vaccine decision-making suggests that the perceived risk posed by the disease to be prevented is weighed against the perceived risk of the vaccines[1-4]. The newspaper coverage of Goody's illness may also have contributed to raising awareness of possible ways to prepare children for the death of a parent, and some of the presentation of Goody's chemotherapy-induced alopecia may contribute towards a more positive image that bald can be beautiful, although it may have reinforced existing negative views about baldness in women[40]. To establish people's awareness and recollection of the coverage and how, in their stories, it has contributed to their understandings of the risks of cervical cancer and need for HPV vaccination, there is a need for qualitative research with key audience groups.

Our study has limitations. It only considered coverage in 15 newspapers, and was not able to examine other popular media (e.g. celebrity magazines, 'fly-on-the-wall' reports of her experiences on commercial television channels) which may have covered different topics or been more widely accessed by young women. Our analysis of coverage ran up to the date of Goody's funeral, and it is plausible that any later coverage or her illness may have emphasised different aspects of the causes, consequences and experience of cervical cancer. Furthermore, it was not designed to examine audience reception and understanding of the coverage.

The extent to which any 'Goody Effect' contributes to saving lives in the longer term depends on how long her story acts as a reminder of the potentially devastating impact of this disease to all women including those in the highest risk groups, and whether it motivates 'hard to reach' groups to believe that actions such as attending for cervical screening and follow up treatments can make the difference between life and death. However, the failure of newsprint coverage of her illness and death to include basic information on the signs and symptoms of cervical cancer, common risk factors for the disease, and the possibility of protecting young women against contracting the viral agent which causes cervical cancer through vaccination presents an ongoing challenge to cancer researchers and specialists in cancer education. Lewison et al highlight cancer researchers' increasing reliance on the "diverse media for informing and engaging the public". They suggest that "If the aim is to achieve a 'public understanding of cancer', then we need to ask how this current interface works and whether it is achieving the aspirations of both the public and the research community"[6]. Our analysis suggests that the widespread interest in and coverage of Goody's illness and death perhaps represents a missed opportunity fully to inform and engage those women who are most at risk of the disease and to mobilise information that could be appreciated more widely by all members of the public.

## Competing interests

The authors declare that they have no competing interests. The Medical Research Council (MRC) funded this research. SH and KH are funded by the MRC.

## Authors' contributions

SH, KH participated in the design, analysis of the data and to the writing and redrafting the final version of the manuscript.

## Pre-publication history

The pre-publication history for this paper can be accessed here:

http://www.biomedcentral.com/1471-2458/10/368/prepub
